# Recognizing the elbow prosthesis on conventional radiographs

**DOI:** 10.1007/s11751-016-0263-5

**Published:** 2016-09-23

**Authors:** Kamilcan Oflazoglu, Nienke Koenrades, Matthijs P. Somford, Michel P. J. van den Bekerom

**Affiliations:** 1 Massachusetts General Hospital, 55 Fruit Street, 02114 Boston, United States; 2 Department of Orthopaedic Surgery, Medisch Spectrum Twente, Haaksbergerstraat 55, 7513 ER Enschede, The Netherlands; 3 Department of Orthopaedic Surgery, Onze Lieve Vrouwe Gasthuis Amsterdam, Oosterpark 9, 1091 AC Amsterdam, The Netherlands

**Keywords:** Elbow, Arthroplasty, Prosthesis, Radiograph

## Abstract

The objective of this study was to make an overview that can be useful in determining which type and brand of prosthesis a patient has when visiting the emergency department or outpatient clinic with a periprosthetic fracture, dislocation, or implant failure. The commonly used prostheses in Europe are opted for this list. The radiographs used for this list are obtained either from the company or from our own patients. This list contains the Coonrad/Morrey total elbow prosthesis, the Nexel total elbow prosthesis, the GSB III Elbow Prosthesis, the iBP Total Elbow System, the Discovery Elbow System, the NESimplavit Elbow System, the Latitude Elbow prosthesis, the Solar Elbow, and the Souter–Strathclyde total elbow. The characteristics of each prosthesis are described.

## Introduction

 With the rising incidence of performing total elbow arthroplasties, orthopedic surgeons and radiologists will more often be confronted with elbow arthroplasty radiographs. The incline in the number of performed total elbow arthroplasties is mainly in acute and post-trauma cases, with probably a decline of the number in rheumatoid elbows because of the high-quality conservative treatment for this disease and, especially in cases of failure of an elbow arthroplasty, because the long-term follow-up of the elbow arthroplasty in general is not similar to the total hip prosthesis. In the Denmark arthroplasty register, an overall 10-year survival of 81 % (95 % CI 76–86 %) was reported [1].

When confronted with a periprosthetic fracture or (a)septic prosthesis failure, a basic knowledge of the implant and type of fixation is useful and probably essential in planning treatment. When no information is available concerning the first operation, the radiograph can guide in recognizing the type and brand of prosthesis. Also knowing whether it concerns a constrained or a non-constrained type helps in identifying hinge failure or recognizing a dislocation.

We present a list of commonly used elbow arthroplasties in Europe with their main features and a lateral radiograph to help the caregiver with identification and subsequent decision making.

## Materials and methods

For this list of elbow prostheses, we opted for the commonly used prostheses in Europe. This list contains the prostheses of which we managed to collect conventional radiographs. The radiographs used for this list are obtained either from the company or from our own patients.

This list contains the next prostheses:

- Coonrad/Morrey total elbow
- Nexel total elbow
- GSB III Elbow Prosthesis
- Discovery Elbow System
- Kudo type-5 prosthesis
- iBP Total Elbow System
- NESimplavit Elbow System
- Latitude Elbow
- Solar Elbow
- Souter–Strathclyde total elbow

## Results

### Coonrad/Morrey total elbow

**Fig. 1:**
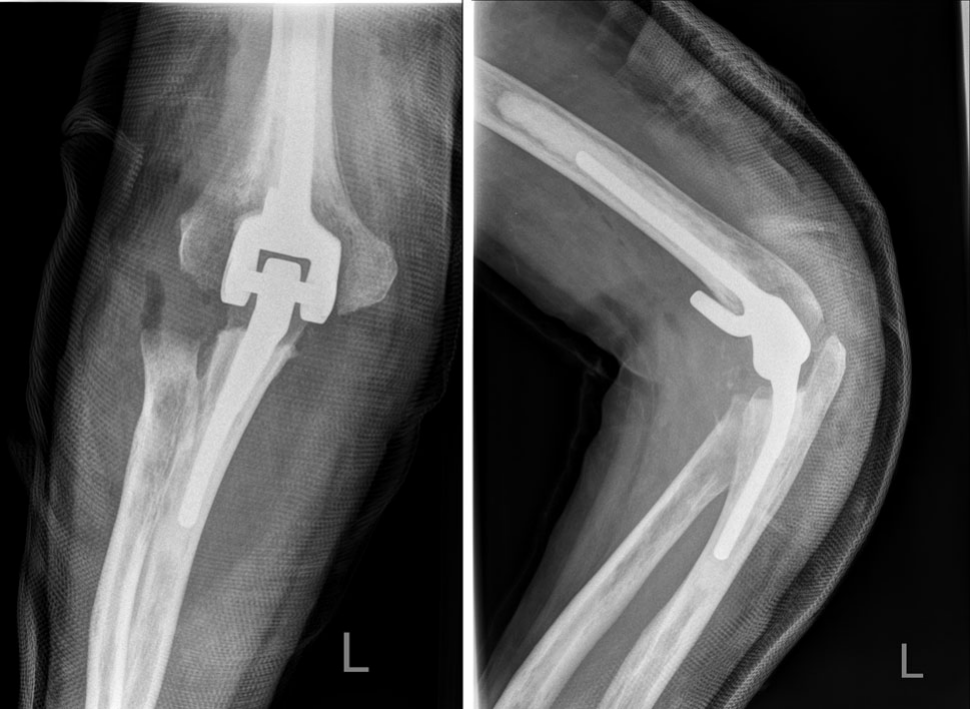
Coonrad/Morrey total elbow; the humeral component of the prosthesis has a humeral anterior flange. The joint surface of the humeral component is quite angular, best seen on the AP view

The Coonrad/Morrey total elbow is produced by Zimmer (Warsaw, IN, USA) as a prosthesis replacing the elbow. The prosthesis is made of Tivanium Ti–6Al–4V alloy and is a cemented prosthesis. The connection of the components is linked, but semi-constrained with a metal–polyethylene bushing. Length of humeral and ulnar components can be varied. The humeral stem is triangular and the ulnar stem is quadrangular. There are 12 different sizes for both the humeral and the ulnar stem. The Coonrad/Morrey total elbow has a 12-year survival of 92.4 % (Fig. 1) [2].

### Nexel total elbow

**Fig. 2:**
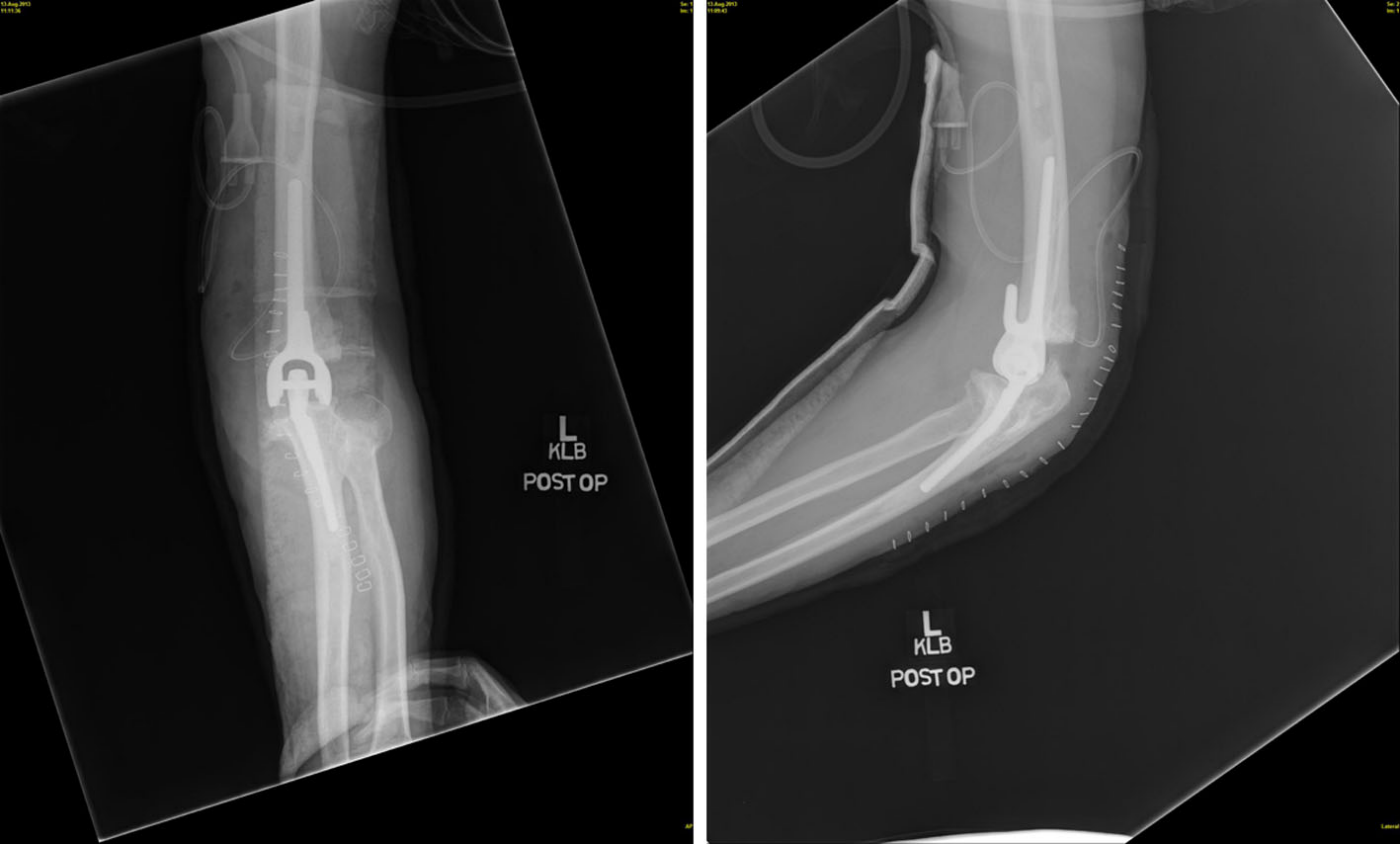
Nexel total elbow; this prosthesis also has a humeral anterior flange. As a result of the ticker bearing, there is more space between the two components on the X-ray compared with the Coonrad/Murray total elbow. The joint surface of the humeral component of the Nexel is more circular shaped, in contrast to the Coonrad/Morrey total elbow, best seen on the AP view

The Nexel total elbow is also produced by Zimmer as a prosthesis replacing the elbow. It is built on the foundation of the Coonrad/Morrey total elbow. The prosthesis is made of Tivanium Ti–6Al–4V alloy and is cemented. The connection of the components is constrained with a different, thicker polyethylene bearing (Vivacit-E HXPE) compared with the Coonrad/Morrey total elbow. Therefore, it may reduce edge loading and stress and maximizes contact area to distribute joint reaction forces. Length of humeral and ulnar components can be varied similar to the Coonrad/Morrey. The intramedullary stem geometry and anterior humeral flange are maintained from the Coonrad/Morrey total elbow (Fig. 2) [3].

### GSB III Elbow Prosthesis

The GSB III Elbow Prosthesis is also produced by Zimmer. The prosthesis is made of titanium alloy and is cemented. The connection of the components between the humeral stem and the ulna component is “plug-in,” non-constrained.

**Fig. 3:**
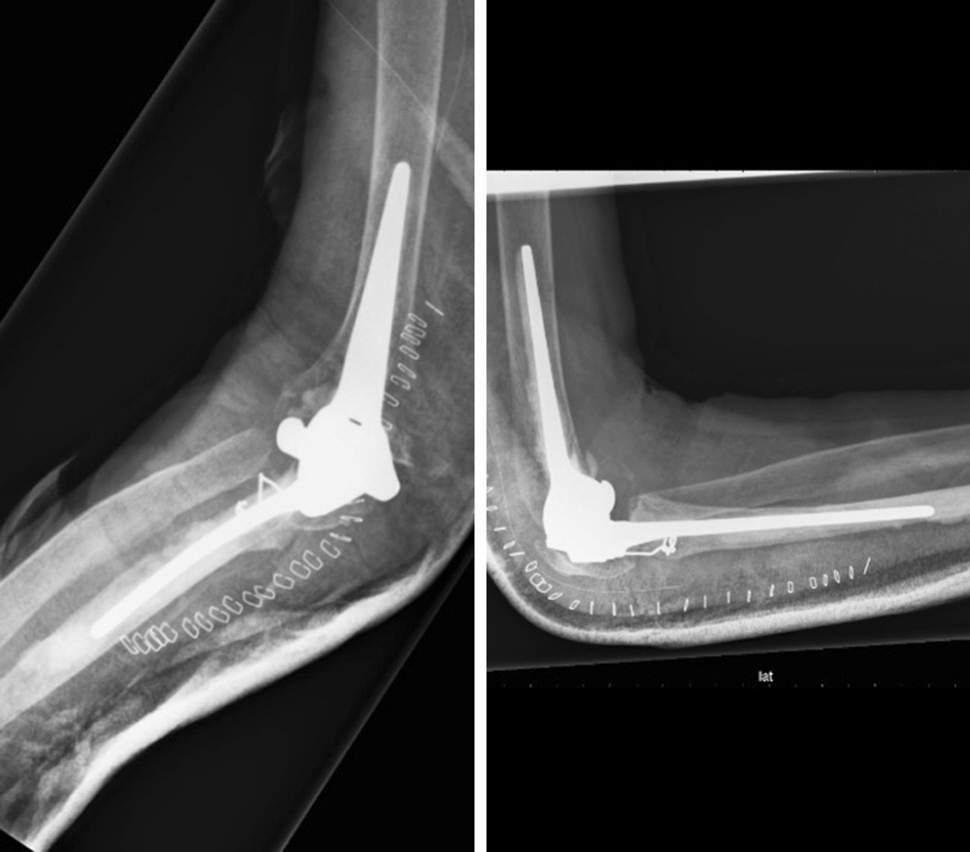
GSB III Elbow Prosthesis; this prosthesis does not have a humeral anterior flange. On an anterior–posterior X-ray, the GSB III Elbow Prosthesis is recognizable because of the large joint surface of the humeral component

The GSB I Elbow Prosthesis was introduced in 1971. At that time, all rigid hinged arthroplasties showed a high rate of loosening. As a result, the GSB III Elbow Prosthesis was developed and is used since 1978. The humeral component has a large surface for support on the condyles and a wide stem for transference of rotational stress. All articulating surfaces are coated with polyethylene. The GSB III Elbow Prosthesis has three humeral sizes and four ulnar components all of which can be freely combined with each other (Fig. 3) [4].

### Discovery elbow system

**Fig. 4:**
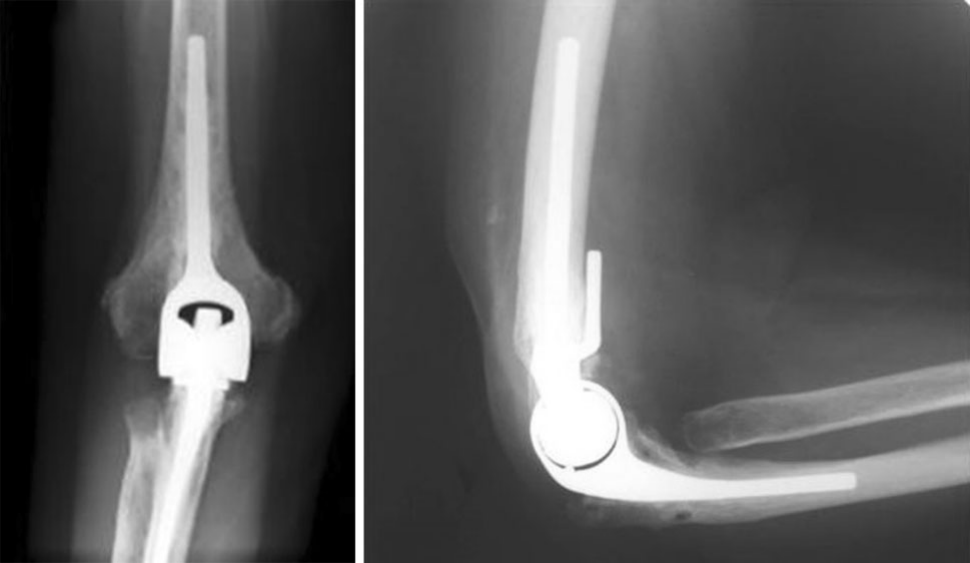
Discovery elbow system; the humeral component of the prosthesis has a humeral anterior flange. It has a characteristic hinge, on de AP view, similar to shape of a horizontal hourglass

The Discovery Elbow System is produced by Biomet (Warsaw, IN, USA). The ulnohumeral prosthesis is made of CoCrMo alloy or titanium alloy, and both components are cemented. The connection of the components is constrained with an ultra-high molecular weight polyethylene (UHMWPE). Length and width of humeral and ulnar components can be varied (Fig. 4) [5].

### Kudo type-5 prosthesis

**Fig. 5:**
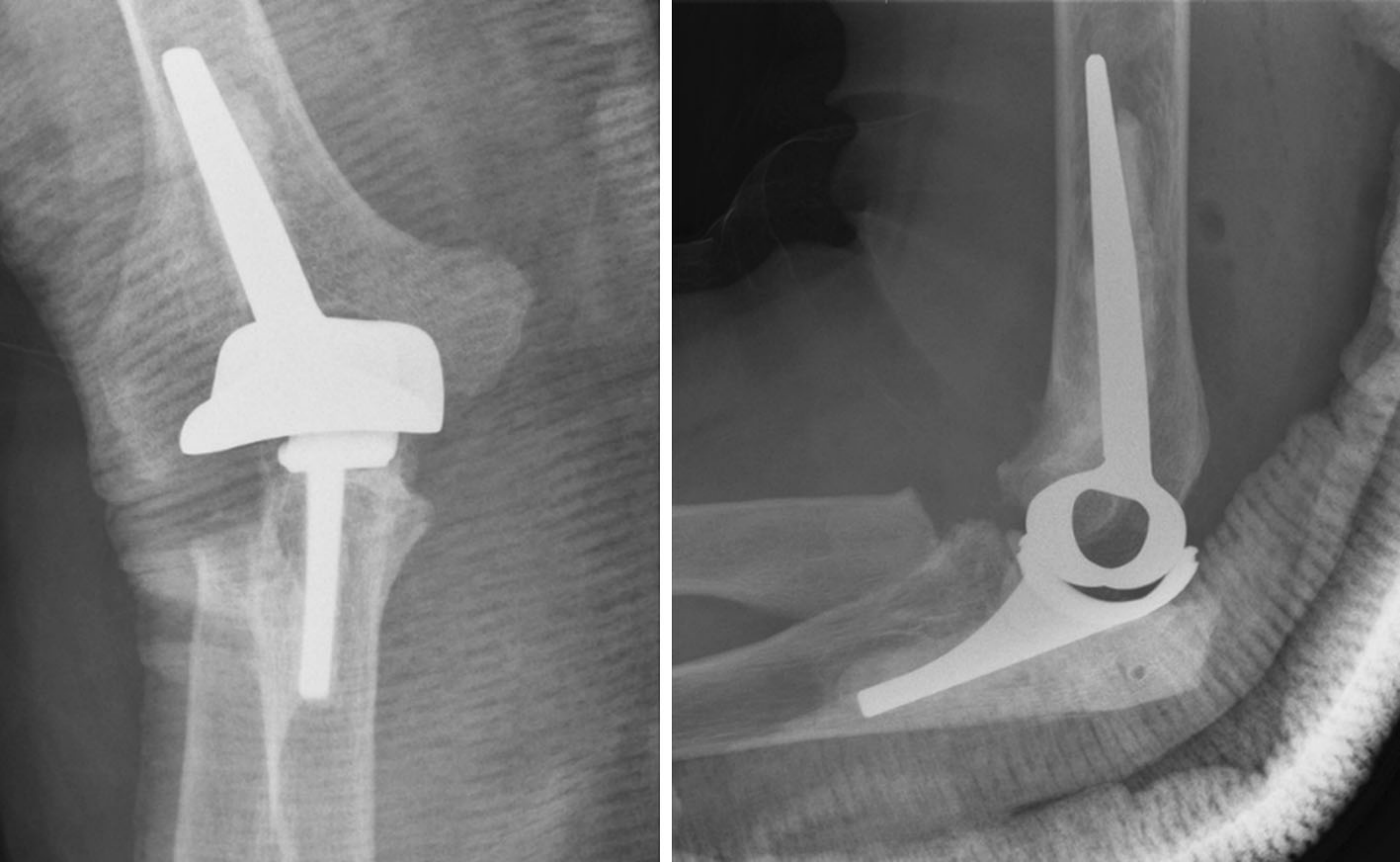
Kudo type-5; both components have a straight and narrow humeral component on an AP view. The tube shape of the distal part of the humeral component, the ‘oculus’, is best seen on a lateral view

The Kudo type-5 prosthesis is produced by Stryker Howmedica Osteonics (Limerick, Ireland) and is a non-constrained, unlinked prosthesis. Contrary to almost every prosthesis on this list, the Kudo type-5 does not require acrylic cement for fixation. The humeral component consists of cobalt–chromium alloy with half of the surface of the stem porous-coated with a plasma spray of titanium alloy. Porous coating of the stem with titanium alloy should achieve osseointegration at the bone–metal interface. The ulnar component either has a metal backing with a porous-coated stem or is either all-polyethylene. In the last case, cement is required for the fixation of the ulnar component. Both components articulate on a high-density polyethylene layer. The stem of both components, especially the ulnar component, are narrow and straight. The distal part of the humeral component is tube shaped (Fig. 5) [6].

### iBP Total Elbow System

**Fig. 6:**
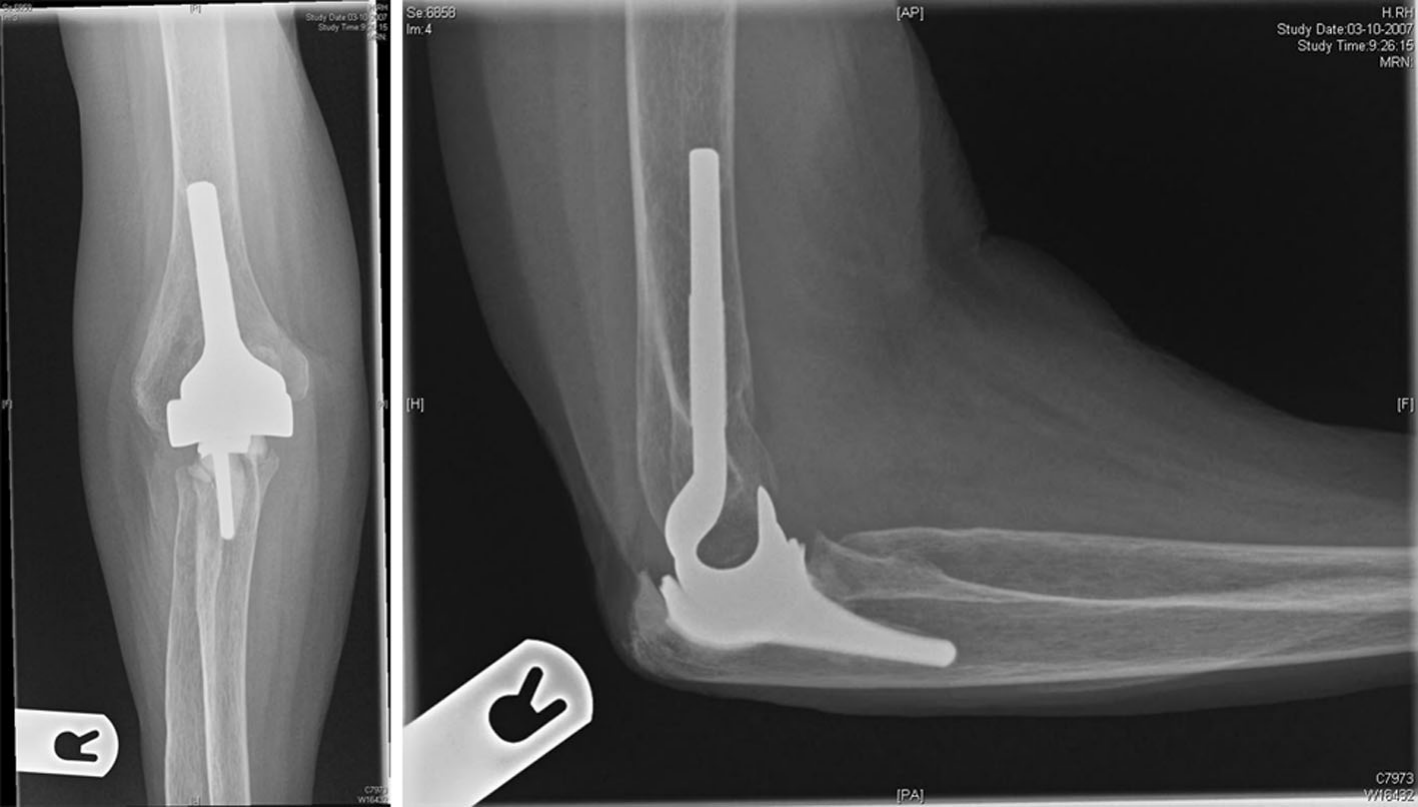
iBP Total Elbow System; there is no humeral anterior flange. This prosthesis has a characteristic hook-shaped humeral component, best seen on the lateral viewed radiograph. The radial component is relatively short

The iBP (instrumented bone preserving) Elbow System is also produced by Biomet (Warsaw, IN, USA). This is a modification of Stryker’s Kudo elbow prosthesis, with more available sizes, improved humeral condyle requiring less removal of humeral bone, and a more anatomical shape. This true unlinked prosthesis has four different humeral components (small, standard, large, and extra large) and three ulnar components (small, standard, and large). Both components are available in uncemented and cemented options. The humeral component is cobalt–chrome and the ulnar component is made of titanium. The articulation is ArCom polyethylene (Fig. 6) [7].

### NESimplavit elbow system

**Fig. 7:**
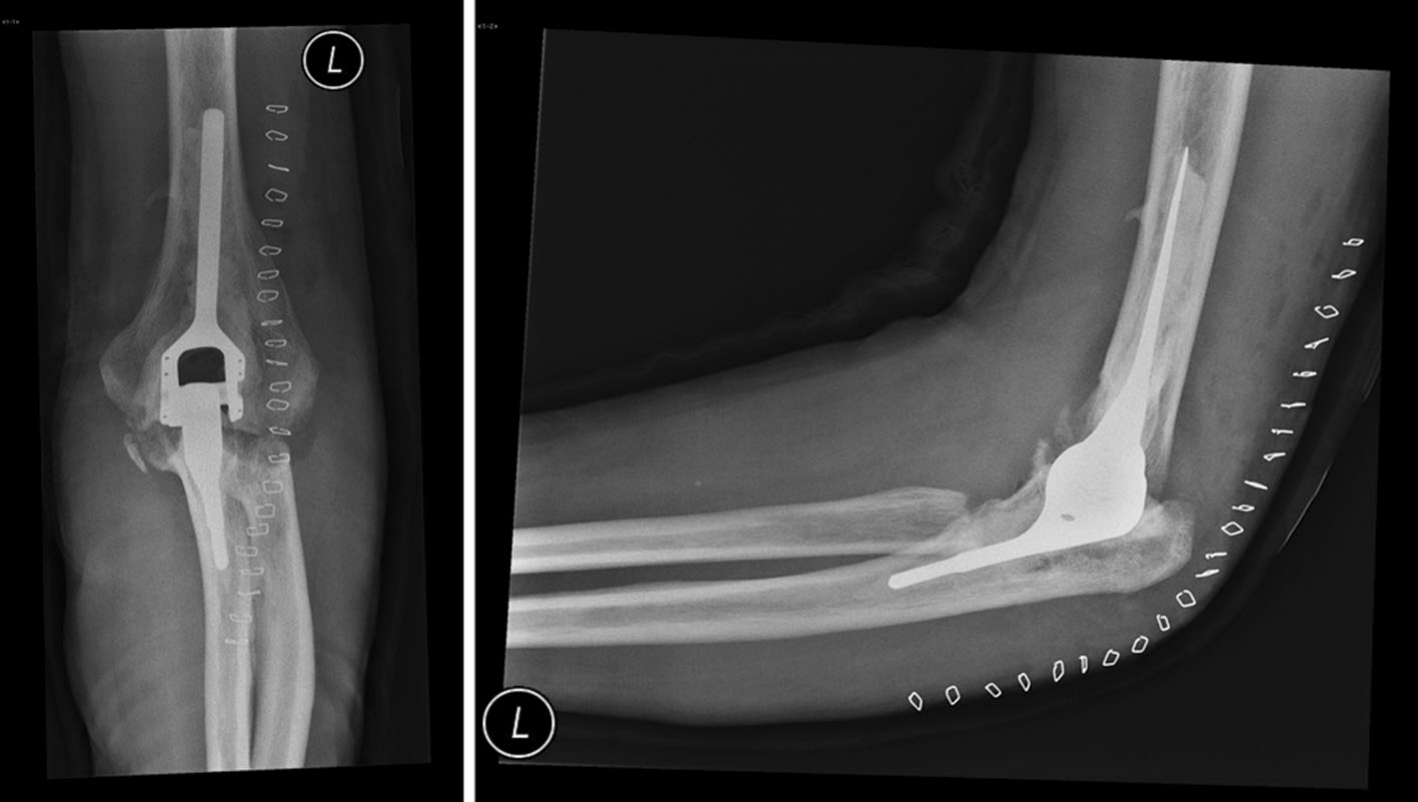
***NES***implavit elbow system; the humeral component of the prosthesis does not have a humeral anterior flange. On the lateral view, the proximal part of the humeral component is smaller than the distal part. The ulnar component is relatively small

The NESimplavit Elbow System, formerly known as the Norway elbow, is produced by Implant Cast (Buxtehude, Germany) as a prosthesis replacing the elbow. Ulnar and humeral components are made of cast CoCrMo alloy. The bobbin is made from UHMWPE. This is the cylinder part of the humeral component which articulates with the ceramic coated axle of the ulnar component. The axle is made from TiAl_6_V_4_ which is coated with TiN to reduce the polyethylene wear. This system is for cemented use only. The connection is semi-constrained. The intact ligaments and tendons are stabilizing the joint. Four different sizes are available for the humeral implants and can be combined independently with the three ulnar sizes (Fig. 7) [8].

### Latitude Elbow

**Fig. 8:**
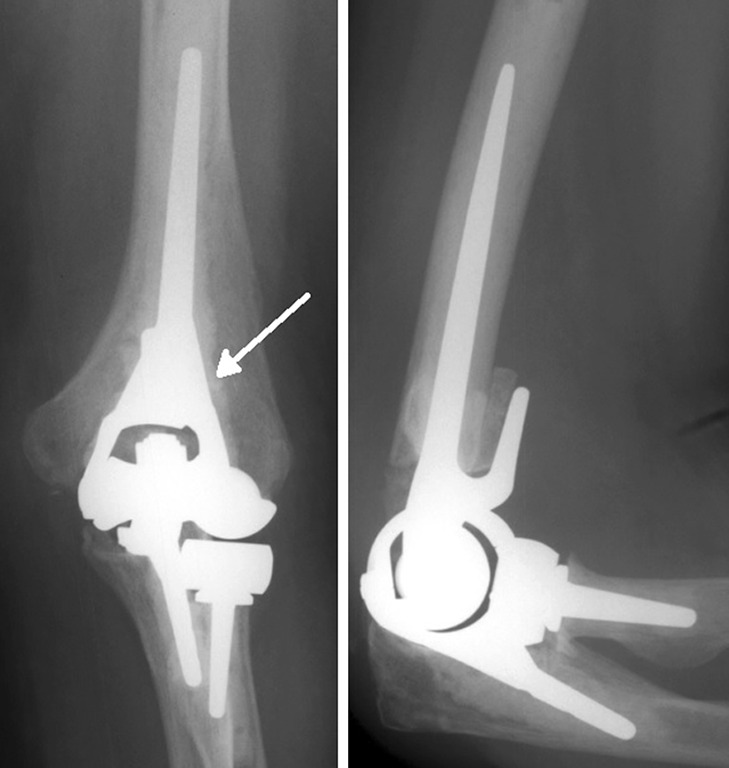
Latitude Elbow; the humeral component of the prosthesis does not have a humeral anterior flange. The medial and lateral fins of the humeral component (best seen on AP view) make the distal part look pyramid-shaped. Noticeable is the radial head component

The Latitude Elbow is produced by Tornier (Montbonnot Saint Ismier, France) as a cemented cobalt–chrome prosthesis replacing the elbow with the possibility of placing a radial component. The Latitude ulnar stem is designed with an optional cap so that the components can be unlinked or linked, constrained, and non-constrained. The ulnar component, as well as the radial head component, articulates with a polyethylene layer. The humeral stem has medial and lateral fins to prevent intramedullary rotation. Humeral spools have been designed with a concave barrel shaped trochlea to preserve linear contact throughout 7 of valgus/varus movement with the ulnar component. In case of a humeral fracture, it is possible to replace solely the humeral component (Fig. 8) [9].

### Solar elbow

**Fig. 9:**
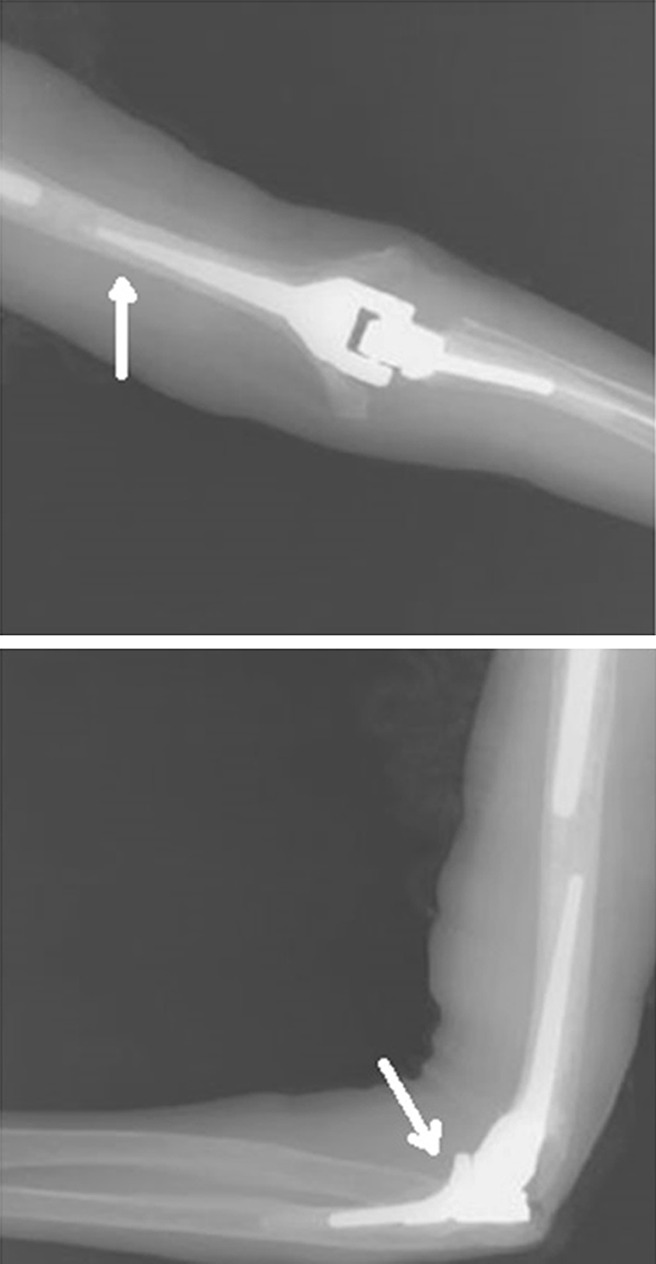
Solar Elbow; the humeral component of the prosthesis does not have a humeral anterior flange. The hinge resembles the Coonrad/Murray on an AP view. However, the proximal paNESimplavit’s humeral component. The subtle antrt of the humeral component is pointy, in contrast to that of the Coonrad/Morrey’s and the erior fin of the ulnar component is seen on the lateral view

The Solar Elbow is produced by Stryker (Kalamazoo, MI, USA) as a cemented titanium alloy prosthesis replacing the total elbow. This linked prosthesis is semi-constrained. The humeral and ulnar component both articulate with a polyethylene layer. Similar to the Latitude Elbow, the humeral stem has medial and lateral fins to prevent intramedullary rotation. The ulnar component has a subtle anterior fin to help resist rotational forces placed across the joint. There are two sizes of humeral components, standard and large, along with three sizes of ulnar components, small, standard, and large (Fig. 9) [10].

### Souter–Strathclyde total elbow

**Fig. 10:**
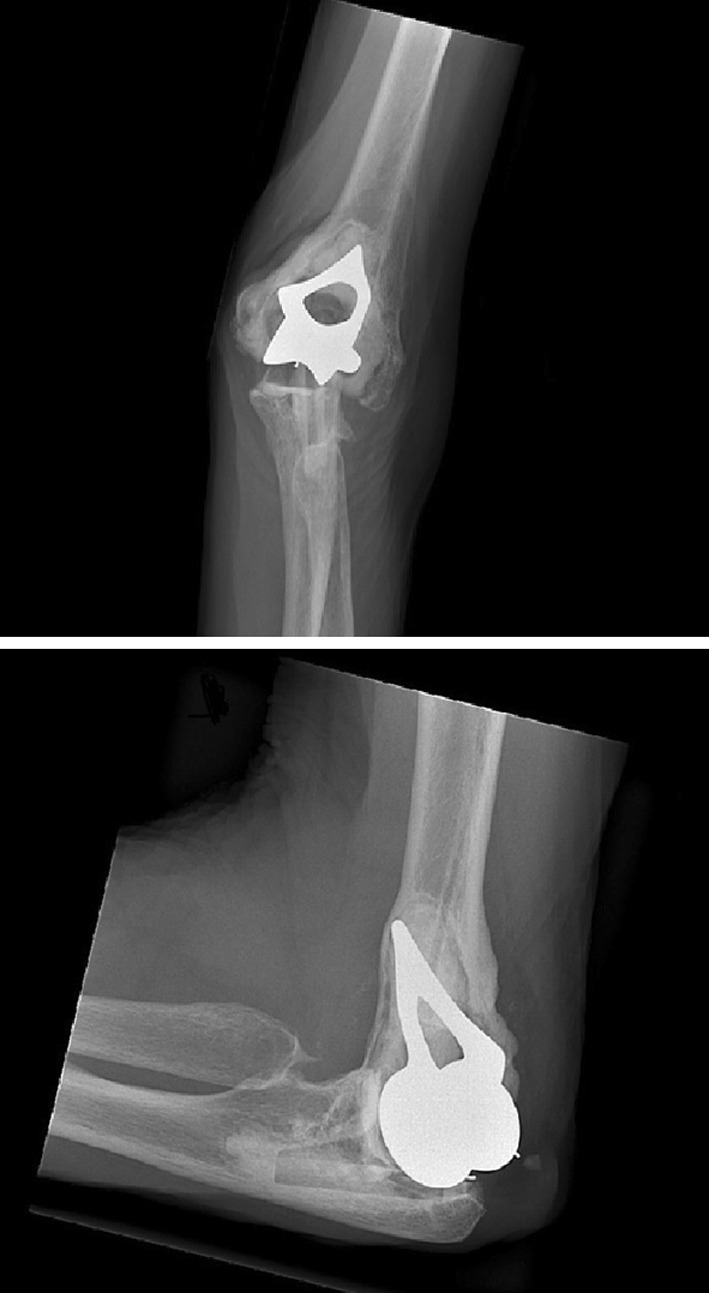
Souter–Strathclyde; the humeral component differs from the other prostheses in this list. On an AP view, the proximal part of the humeral component has a pointed shape with a gap in the middle. It is relatively short. The ulnar component is less visible

The Souter–Strathclyde total elbow is produced by Stryker Howmedica Osteonics (Limerick, Ireland) is a cemented unlinked and partially constrained elbow prosthesis. This elbow prosthesis differs from the other prostheses in this list because of the characteristic humeral component with humeral flanges projecting into the capitellum and the medial epicondyle. The relatively short humeral component is made of Vitallium is available in a small, medium, or large size. It is also available with long stems. The ulnar component has a keel and a small stem, and is made of ultra-high molecular weight polyethylene, which, at first sight, makes it less visible on a radiograph (Fig. 10) [11–13].

## Discussion

The number of total elbow arthroplasties is growing in (post-) trauma cases. Although several imaging modalities are available, conventional radiography remains the mainstay of imaging evaluation of elbow replacements. The various types and brands of elbow replacements can be recognized on radiographs. In this article, an overview was provided of the most commonly used elbow arthroplasties in Europe and their specific characteristics.

This list contains the Coonrad/Morrey total elbow prosthesis, the Nexel total elbow prosthesis, the GSB III Elbow Prosthesis, the Discovery Elbow System, the Kudo type-5, the iBP Total Elbow System, the NESimplavit Elbow System, the Latitude Elbow prosthesis, the Solar Elbow, and the Souter–Strathclyde total elbow.

**Fig. 11:**
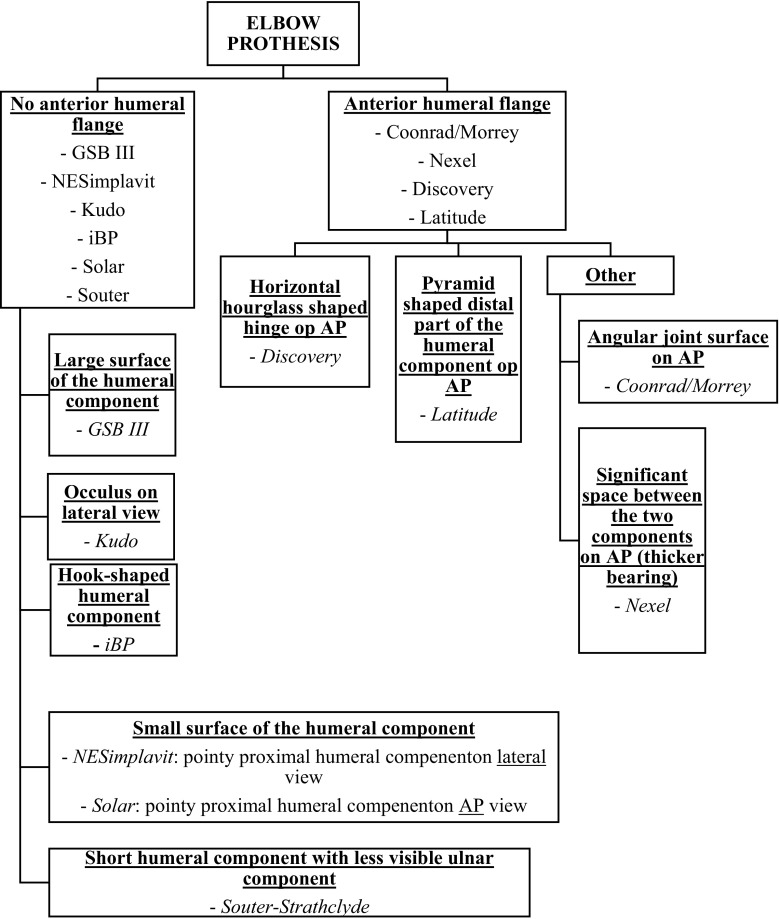
Characteristics of each elbow prosthesis

An important recognizable part of elbow prostheses on a lateral radiographic view is whether the humeral component of the elbow prosthesis has an anterior humeral flange. In this list, the Coonrad/Morrey total elbow prosthesis, the Nexel total elbow prosthesis, the Discovery Elbow System, and the Latitude Elbow prosthesis have a humeral anterior flange (Fig. 11).

Of all the prostheses on this list, only Tornier’s Latitude Elbow prosthesis has a radial head component. All of the prostheses described in this overview are for cemented use, except for the Kudo type-5 and the iBP Total Elbow System (that provide both options). They all come in different sizes.

The Latitude Elbow can be used as a both constrained and non-constrained prosthesis, depending on the ability of the surrounding joint structures to provide stability to the joint.

The Souter–Strathclyde total elbow has a characteristic humeral component with a less visible ulnar component on a conventional radiograph.

## Conclusion

This overview can be useful in determining which type and brand of prosthesis a patient has when visiting the emergency department or outpatient clinic with a periprosthetic fracture, dislocation, or implant failure.
